# Comparative analysis of different PV technologies under the tropical environments

**DOI:** 10.1038/s41598-025-99958-x

**Published:** 2025-05-11

**Authors:** V. Femin, R. Veena, M. I. Petra, S. Mathew

**Affiliations:** 1 Universiti Brunei Darussalam, Jalan Tungku Link, Gadong, BE 1410 Brunei Darussalam; 2 Faculty of Engineering and Science, University of Agder, Jon Lilletunsvei 9, 4879 Grimstad, Norway

**Keywords:** Solar energy, Array yield, Reference yield, Capture loss, Performance ratio, Efficiency ratio, Power ramps, Generalized logistic distribution, Machine learning, ANN, SVM, kNN, Energy science and technology, Engineering

## Abstract

In this paper, six different types of solar PV technologies are compared in terms of their performances under tropical conditions, using three years of performance data from a 1.2 MW experimental solar farm. The technologies considered include single-crystalline silicon, polycrystalline silicon, microcrystalline silicon, amorphous silicon, copper indium selenium (CIS), and hetero-junction with intrinsic thin layer (HIT). The field performances of these cells were initially assessed using standard performance indices such as Array Yield, Reference Yield, Capture Loss, Performance Ratio, and Efficiency Ratio. Among the technologies studied, amorphous silicon and HIT-based systems demonstrated better performance, showing higher Performance and Efficiency Ratios, along with lower capture losses. This study also modelled the fluctuations in power production from these panels. Under probabilistic modeling, the ramping behavior of the systems was characterized using the Generalized Logistic Distribution. Based on this analysis, CIS PV systems were found to have minimum power ramps, where as the HIT based systems showed the highest power fluctuations. To predict minute-wise and hourly ramping of the PV systems under varying levels of solar insolation, machine learning methods based on Artificial Neural Networks (ANN), Support Vector Machines (SVM), and k-Nearest Neighbors (kNN) were developed. With a Normalized Root Mean Square Error (NRMSE) of over 96%, these models demonstrated high accuracy in capturing the ramping characteristics of the studied PV systems. The results of this study offer valuable insights into the performance of different PV systems under tropical regions, which can be used in efficiently designing and managing solar PV projects.

## Introduction

Owing to its tremendous resource potential, a significant increase in system efficiencies and a constant decline in the system cost, the global installed capacity of solar PV systems has significantly increased in recent years. For example, with an added capacity of 446 GW during the year, the global solar PV installations have reached around 1581 GW by 2023, which is sufficient for 5.5% of global energy consumption^1,2^. The future prediction indicates that, by 2050, the cumulative installed capacity around the world will exceed 8519 GW which contribute up to 25% of the global energy consumption^3^. Most of these installations are planned to be grid-connected. With this increasing global presence, solar PV technologies have been considered as one of the major contributors to the future clean energy scenario.

There are various types of solar PV technologies, each differing in materials and structural features. The efficiencies of these systems also vary significantly^4^, which in turn impacts on the cost of generation. Hence, understanding the comparative performance of different PV technologies under the operating environment of a prospective project is crucial for planning and developing a successful solar PV program.

Similarly, one of the major challenges that the power community may face with large PV installations is the frequent fluctuations in the power produced by solar PV plants. Due to the stochastic nature of solar irradiance, power output from the solar plants would vary significantly with time. This may pose significant challenges to power system operators and planners as sufficient reserves, with quick ramping-up capabilities, are to be made readily available to handle these power fluctuations. Hence, a better understanding of these performance variations of the systems and the corresponding power ramps is essential for the efficient design and management of the PV-integrated power systems.

In recent years, several studies have been conducted to understand the short-term output fluctuations of solar PV systems^5–21^. Most of these studies adopt statistical approaches under which the Probability Density Function (PDF) and Cumulative Distribution Function (CDF) of the power variability are used to define the solar power fluctuation^13,15,18,20,21^ In another attempt, transfer functions based on Discrete Fourier Transform (DFT) and Fast Fourier Transform (FFT) algorithms are used for predicting the power output fluctuations^9,17^. Machine learning methods like Artificial Neural Networks (ANN), Convolution Neural Networks (CNN), Support Vector Machine (SVM), k-nearest neighbor (kNN), and Random Forest (RF) are also being tried in a few studies for analyzing solar power variability^6–8,10,11,14,16,19,22^.

Several studies have investigated the performance of various photovoltaic (PV) technologies in different climatic conditions^23–27^^.^ A study conducted in Hungary reported that polycrystalline silicon (pc-Si) performed well in that region based on a dataset collected over a two-year period with a 10-min resolution^24^. Similarly, based on the performance analysis of five different PV technologies in a humid tropical climate in Ghana, it is concluded that HIT-based systems were the most suitable for space-constrained environments, whereas CIS technology was the least effective. Their study also found that polycrystalline silicon was the most suitable PV technology, followed by amorphous silicon (a-Si) and HIT^27^. In another study in Morocco’s hot semi-arid climate, it was found that monocrystalline silicon (m-Si) was the most efficient, although polycrystalline silicon (p-Si) was more cost-effective, making it the preferred choice for the region^25^.

Further, simulation tools such as PVsyst, PVWATTS, TRNSYS, HOMER, SolarPro, were used for predicting the performance of a 1 MW PV power plant under India’s arid climatic conditions^23^. It is reported that CIS modules yielded the highest annual energy output of 2471 MWh, with a performance ratio (PR) of 86.1%, while mono-Si exhibited the highest annual temperature-related energy loss (14.4%). In addition, evaluation of PV performance under similar outdoor conditions in India reported that a-Si outperformed p-Si during summer due to its thermal annealing effect but underperformed in winter^28^. Meanwhile, HIT consistently showed better performance than p-Si throughout the year. A comparative study conducted at Nanjing, China, assessed multiple PV technologies and identified HIT as the best-performing option, while CdTe and p-Si were the least suitable^26^.

Overall, these studies indicate that performances of PV technologies are highly dependent on the climatic conditions under they operate. Further, though these investigations could provide valuable information about the variability of solar PV systems under changing insolation levels at the study location, most of these studies consider only one specific type of PV technology. Similarly, several of these studies rely on smaller, stand-alone systems with a limited capacity of a few kilowatts. Furthermore, many of these studies rely on data collected during short durations and hence do not fully capture the long-term performance characteristics.

The specific contributions of this paper are as follows: (1) A comprehensive analysis and comparison of the performance and ramping behavior of six different solar PV systems operating at the same site and under identical environmental conditions. (2) Three years of data are utilized for analysis and model development to minimize annual variations and model errors. (3) Data from a 1.2 MW experimental PV power plant is used. The larger plant size, and consequently the higher data volume, enhances the statistical significance of the analysis compared to studies based on smaller systems. This approach also helps average out potential noise in the datasets. Additionally, the larger plant size aids in gaining a better understanding of the systems’ interactions with the grid.

Following the introductory section and a description of the experimental solar PV plant, the results of this study are presented in two parts. In the first part, the performance of six different solar PV technologies is quantified and compared in a representative tropical environment using standard performance metrics. In the second part, the analysis is extended to model the ramping behavior of these systems. This includes the probabilistic modeling of minute-by-minute and hourly ramping, as well as predictive modeling of power ramps using machine learning methods. Such power ramp models can assist system managers in optimizing power dispatch strategies. Furthermore, when these models are integrated with forecasts of solar insolation, they can be useful for plant owners and utilities participating in energy markets.

## The experimental solar power plant

**Fig. 1:**
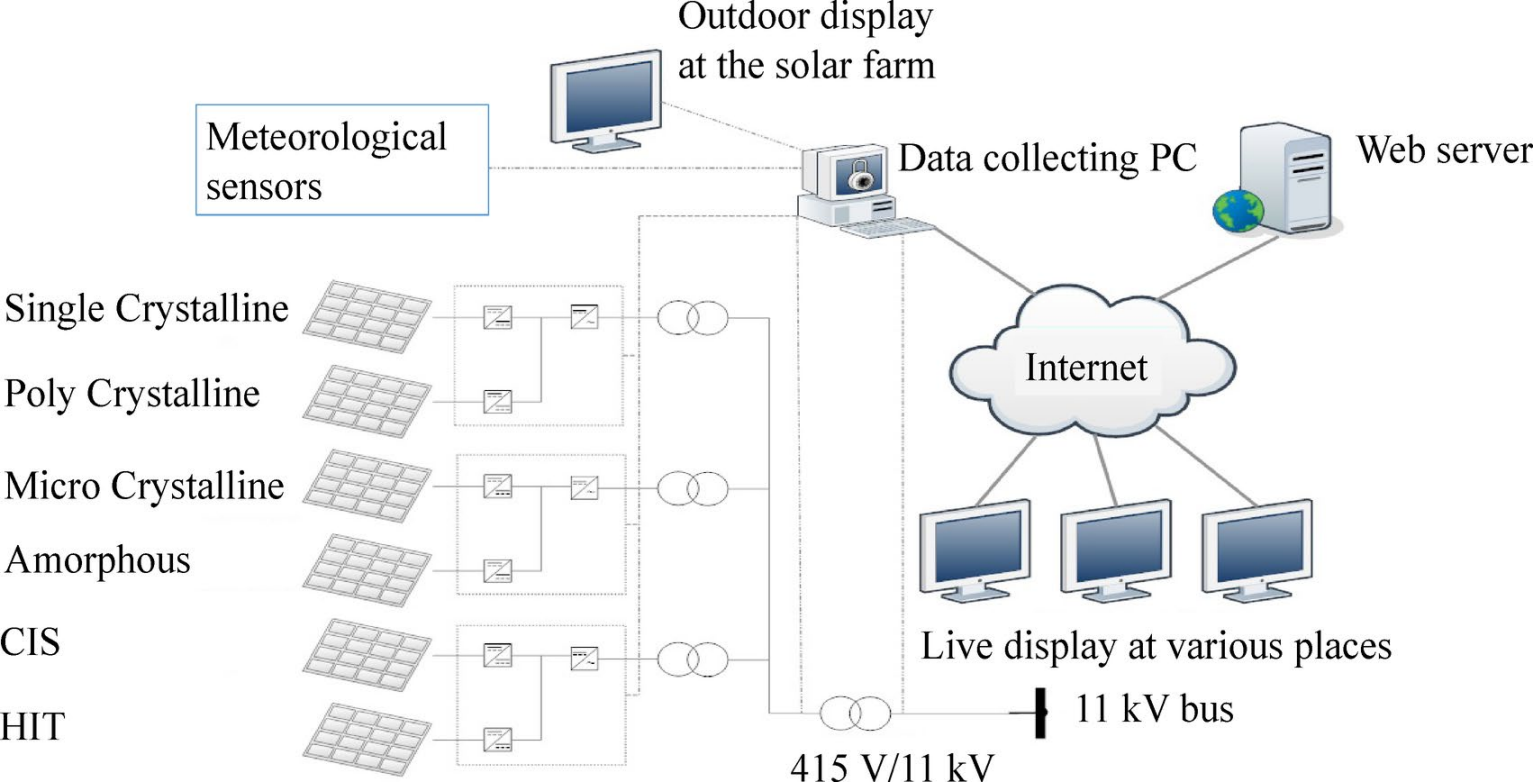
Features of the experimental solar PV power plant.

The solar farm selected for this analysis is the Tenaga Suria Brunei (TSB) power plant situated in Seria, Belait District, Brunei Darussalam. The plant location has a latitude of 4°37′ N and a longitude of 114°23′ E. The configuration of the solar photovoltaic (PV) power plant and the data monitoring system are shown in Fig. 1^22,29^.

The TSB power plant is equipped with six different types solar PV systems, all of which have a rated capacity of 200kWp. The PV types are (1) single crystalline silicon (sc-Si), (2) polycrystalline silicon (mc-Si), (3) microcrystalline silicon (nc-Si/a-Si), (4) amorphous silicon (a-Si), (5) copper indium selenium (CIS), and (6) hetero-junction with intrinsic thin layer (HIT). The modules’ technical specifications are provided in Table 1^22^. As evident, different panels have different conversion efficiencies and hence, the number of modules and the required area for a given rated power are different.

**Table 1:** Solar PV modules’ technical specifications.

Specification	Single crystalline silicon	Polycrystalline silicon	Microcrystalline silicon	Amorphous silicon	CIS (copper indium selenide)	HIT (heterojunction with Intrinsic Thin layer)
Module power output (W)	180	185	130	100	80	205
Efficiency at STC (%)	14.1	13.4	8.2	6.3	8.9	16
Total Number of modules	1116	1098	1540	2000	2500	980
Coverage area (m^2^)	1462	1518	2426	3150	1979	1,25

## Comparative performance of PV systems based on standard metrics

The standard indices like the Array Yield, Reference Yield, Capture Loss, Performance Ratio, and Efficiency Ratio are used to quantify the performance of the PV systems considered in this study. These are briefly discussed below.

### Array yield

The array yield represents how effectively the PV array harvests solar energy and converts it into electrical energy. It measures the actual energy output of the PV array relative to its rated power and is expressed as a ratio calculated on a daily, monthly, or yearly basis. By comparing the actual energy output to the rated power, the array yield provides insights into the system’s performance and efficiency. Understanding the array yield helps to evaluate the performance and economic viability of PV systems, aiding in decision-making regarding system design, installation, and optimization.

**Fig. 2:**
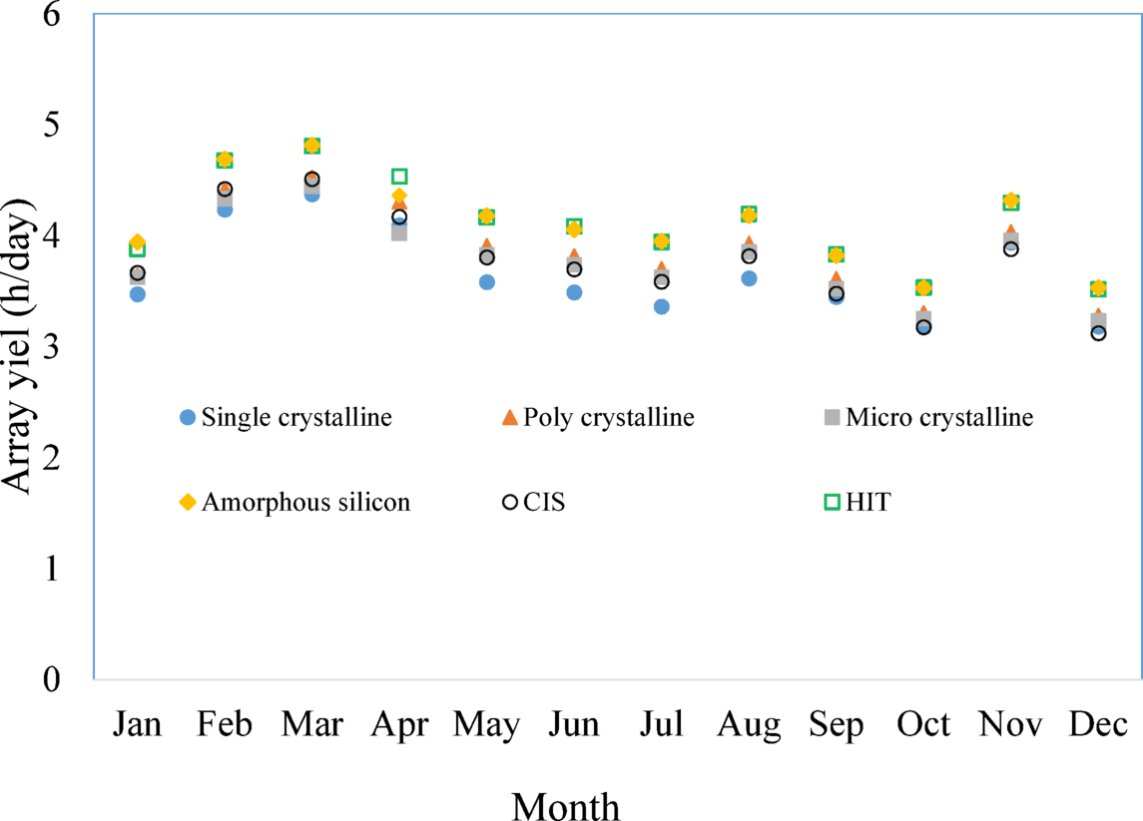
Average daily energy output of various photovoltaic (PV) modules monthly.

Array Yield (*Y*_A_) is calculated using the following equation.

1 $$Y_{A} = \frac{{E_{DC} }}{{P_{0} }}$$

where* E*_*DC*_ is the total DC energy generated by the PV array during the specified period, measured in kilowatt-hours (kWh) and *P*_*0*_ is the rated capacity of the PV system represented in kilowatts peak (kWp). The monthly array yields vary across different PV module technologies and months are presented in Fig. 2. The array yields of these systems vary throughout the year, indicating seasonal variations in energy output. The CIS-PV module system had the lowest array yield of 3.12 h per day in December, while the amorphous silicon PV module system had the highest array yield of 4.81 h per day in March shown in Fig. 2.

### Reference yield

The reference yield (*Y*_*R*_) compares daily in-plane radiation received by PV panels to a predefined reference value to assess PV system performance. It is calculated by comparing the total daily in-plane radiation *(H*_*d*_*)* to the reference radiation *(G)*. By a standardized reference level, the reference yield measures the capacity of PV panels to capture and transform solar radiation into electrical energy. It measures the PV system’s sunlight utilization compared to the ideal 1 kW/m^2^ solar radiation.

The reference yield (*Y*_*R*_) equation is below.

2 $$Y_{R} = \frac{{H_{d} }}{G}$$

Here, *H*_*d*_ is the daily in-plane radiation incident on PV panels in kWh/m^2^ and *G* is the standardized reference value of solar radiation received in the PV panel plane in kW/m^2^.

### Capture loss

Capture loss measures efficiency losses or deviations from ideal performance in a PV system by comparing the reference yield *(Y*_*R*_*)* to the array yield *(Y*_*A*_*).* As shown below, it is the difference between the PV panels’ reference radiation-based expected energy output and the system’s actual energy output.

3 $$L_{C} = Y_{R} - Y_{A}$$

**Fig. 3:**
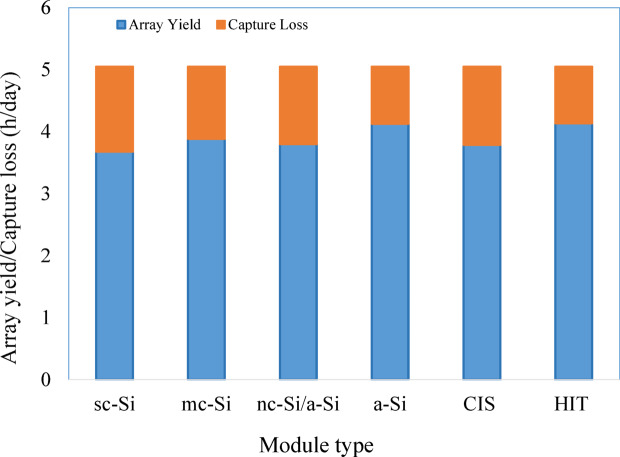
Annual averaged daily array yield and capture losses of different PV modules.

Capture losses of different PV systems are shown in Fig. 3. The lowest capture losses are 18.06% for heterojunction with intrinsic thin layer (HIT) modules followed by 18.22% for amorphous silicon (a-Si) modules. Single-crystalline silicon (sc-Si) modules have the highest capture losses (27.15%). Various factors contribute to capture loss, which diminishes the overall efficiency of the PV array in capturing and converting energy. The difference between expected and actual energy generation could be due to shading, soiling, system degradation, or suboptimal system design. Higher cell temperatures cause thermal capture losses, thereby decreasing cell efficiency.

### Performance ratio

By comparing array yield (*Y*_*A*_) to reference yield (Y_R_), the performance ratio (P_R_) measures photovoltaic (PV) system efficiency. It shows how well the system converts solar energy into electricity. The performance ratio considers shading, soiling, module degradation, and non-ideal conditions to assess system efficiency. It is calculated mathematically as

4 $$P_{R} = \frac{{Y_{A} }}{{Y_{R} }}$$

**Fig. 4:**
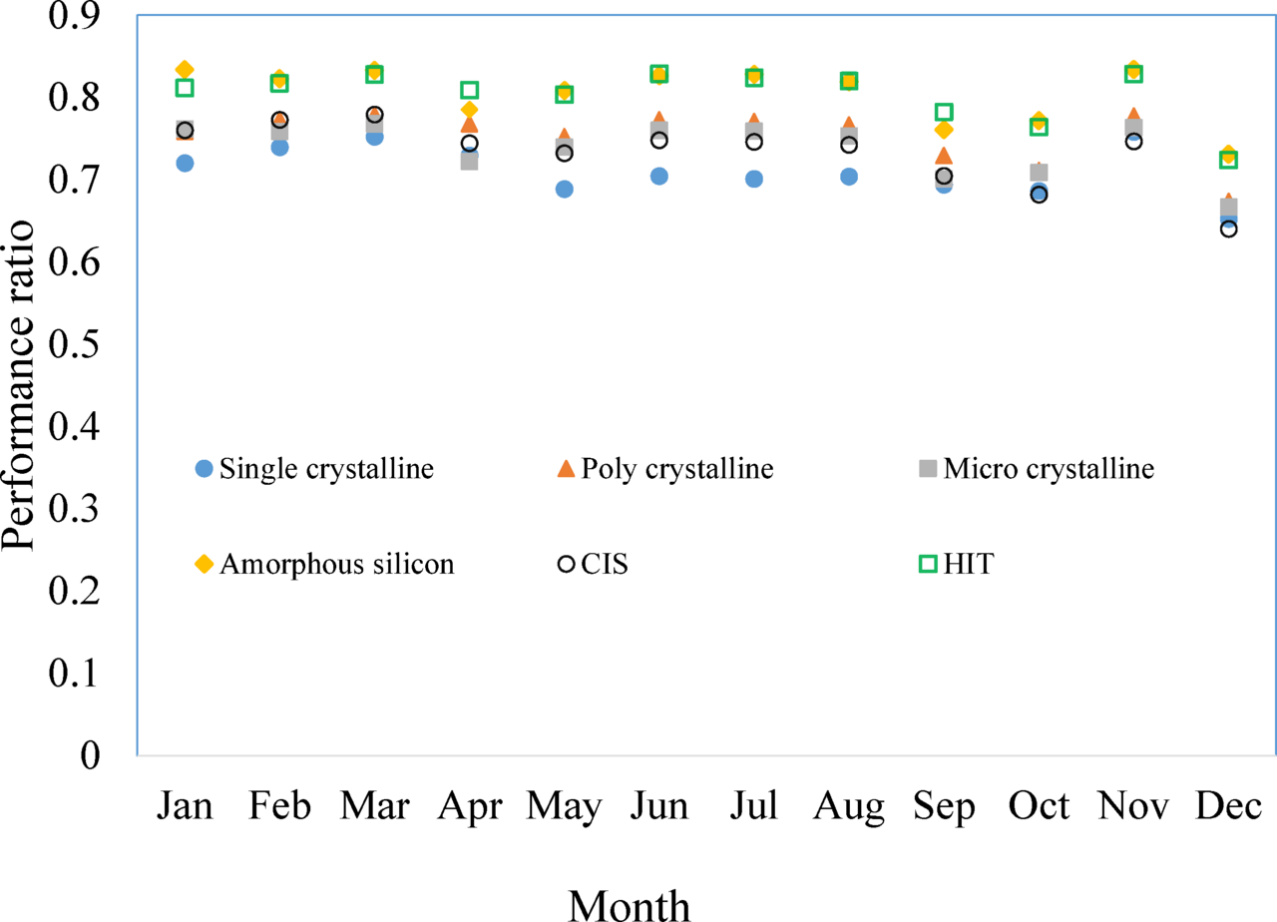
Monthly performance ratios of different PV modules.

A lower performance ratio indicates system losses. Amorphous silicon and HIT PV had the highest monthly performance ratios as shown on Fig. 4. Performance ratios were highest in months with lower ambient temperatures. Indicating the temperature sensitivity of the cells.

### Efficiency ratio

The efficiency ratio (η_R_) is a key factor in determining the field performance of a PV system. It reflects the system’s ability to convert array in-plane irradiation into DC energy output under real-world conditions. The efficiency ratio, which compares energy output (E_DC_) to in-plane irradiation (H) incident on the array, normalized by efficiency at standard test conditions (ηSTC), offer valuable insights into system performance. The efficiency ratio (η_R_) is calculated as:

5 $$\eta_{R} = \frac{{\frac{{E_{DC} }}{H}}}{\eta STC}$$

**Fig. 5:**
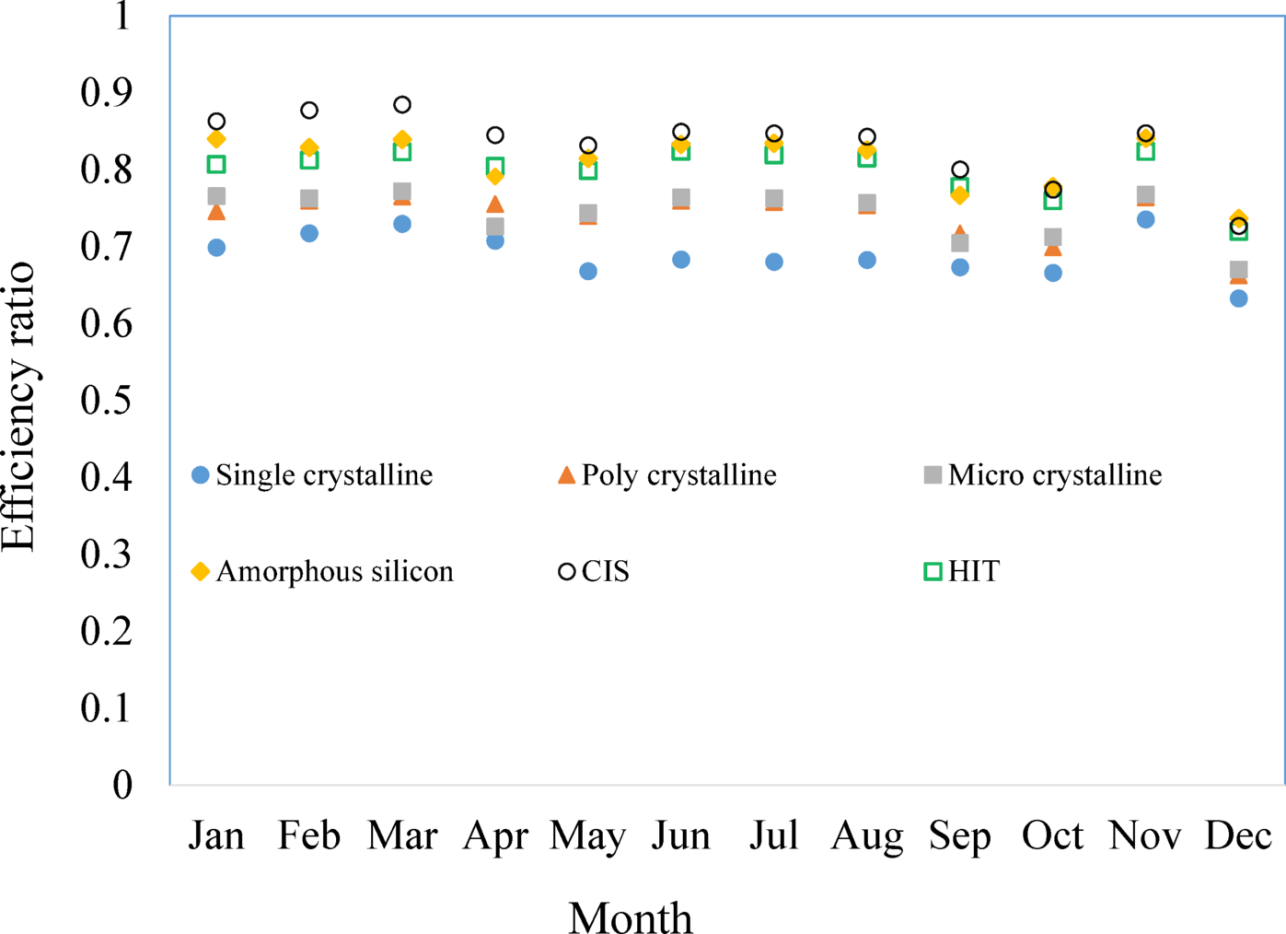
Comparison of the monthly average daily efficiency ratios of different PV modules.

Module efficiency, shading, soiling, and temperature losses are mainly reflected in the efficiency ratio. Figure 5 provides a comparison of the monthly averaged daily efficiency ratios of different PV modules, which indicate how well the PV modules can perform in practical scenarios. From the figure, it is evident that the field performance of CIS modules closely aligns with their rated performance throughout the year. These modules exhibit an annual average efficiency ratio of 0.83, indicating a strong correlation between laboratory and real-world performance. Following this, the amorphous silicon (a-Si) modules could achieve an average efficiency ratio of 0.81, closely followed by HIT modules with a ratio of 0.8. On the other hand, single crystalline silicon (sc-Si) cells exhibited the lowest performance ratio of 0.69.

**Fig. 6:**
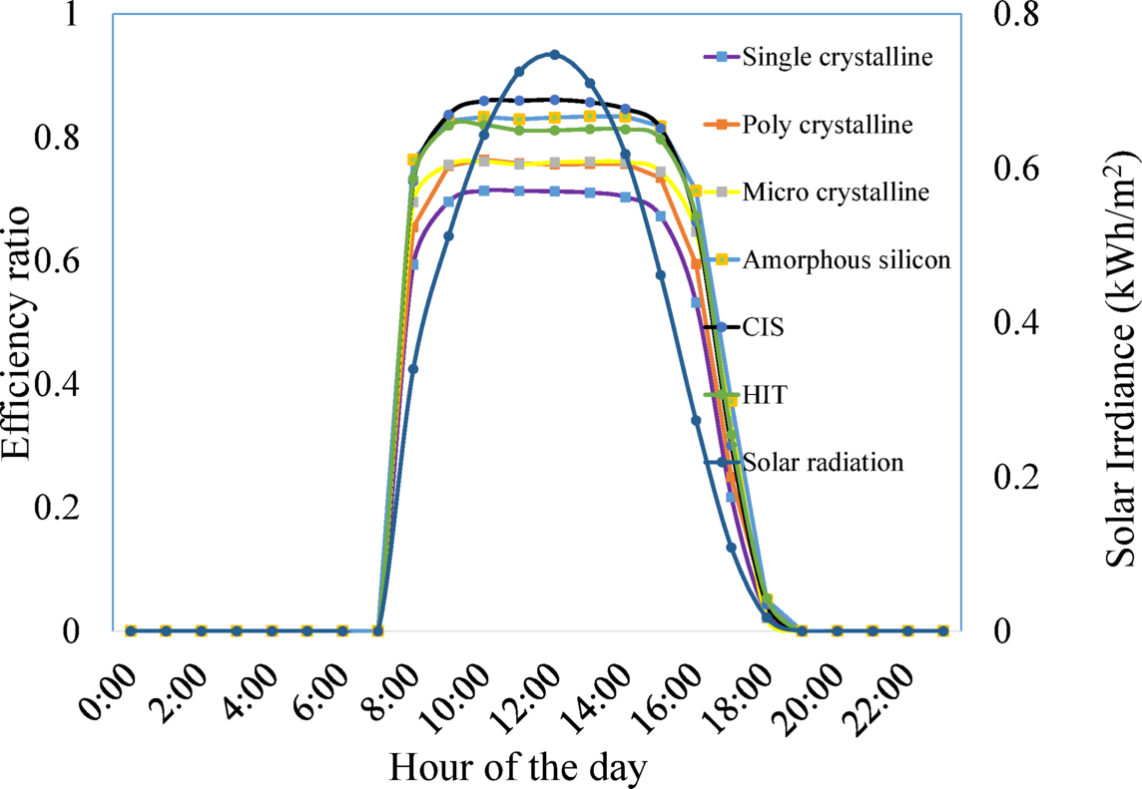
Hourly variations in solar insolation levels and the efficiency ratio.

Figure 6 displays the hourly changes in solar insolation levels and the efficiency ratio of cells on an annual basis. Like crystalline and amorphous silicon, second-generation modules attain their maximum efficiency ratios earlier when exposed to lower levels of insolation. Amorphous silicon and HIT based systems consistently maintain higher efficiency ratios, even in conditions of lower insolation, surpassing other technologies. CIS modules exhibit commendable efficiency ratios, especially in situations with lower levels of solar radiation. The second-generation thin film technologies show relatively better efficiency across a wider range of solar insolation. This adaptability is highly advantageous in regions such as Brunei Darussalam, where frequent cloud cover resulting in substantial variations in solar insolation is prevalent.

## Modelling the ramping behavior of the PV systems

### Data preprocessing and feature selection

To model the ramping behavior of different solar PV systems, power output and environmental parameters like solar insolation, ambient temperature, and wind speed were collected from the experimental solar farm. These data, which are recorded every second, are then averaged to minute and hourly data to examine the ramp rates. The data was cleaned to remove the outliers and noises. Out of the three years of data, two years were used for model training and development, followed by testing with the remaining one-year dataset.

Various weather parameters like ambient temperature, irradiance and wind speed could influence the power production from the PV systems. From the input data considered for this study, the relative influence of these parameters was further analyzed by Gradient Boosted Machine (GBM) algorithm^30^. The results showed that irradiance, with a high correlation of 99.9%, is the major parameter influencing the PV system output, compared to the wind speed (0.07%) and ambient temperature (0.04%). This agrees with the observations in^31^. Data between 8 A.M and 6 P.M have been considered for developing the models as these are the productive hours for the PV systems at the site. The production data are normalized with the watt peak capacity of each panel. The fluctuations in the power output of the systems are defined as

6 $$\Delta P_{o} \,(t)\,\, = \,\,\frac{{P_{o} \,(t + \Delta t)\, - \,P_{o} (t)}}{{P_{R} }}$$

where *ΔP*_*o*_* (t)* is the change in power output at an instant time *t, P*_*o*_ represents the power output, *Δt* is the sampling time interval, and* P*_*R*_ is the total capacity of each panel type (200 kWp).

### Ramp analysis based on the probabilistic approach

The ramp rates of all the PV systems were computed using Eq. 6, which were then fitted with different probability models. The most appropriate distribution, fitting well with the data, were identified using Kolmogorov–Smirnov (K-S) of Goodness of Fit (GOF) test^32–34^. The Kolmogorov–Smirnov (K-S) test assesses the goodness of fit between observed and computed data by comparing the maximum difference in their cumulative distributions. A K-S statistic closer to zero indicates a better fit. Based on the K–S statistic presented in the Table 2, the ramp-up and ramp-down rate of minutes and hourly power fluctuation for all six panels are well described by Generalized Logistic Distribution.

**Table 2:** Goodness of fit statistics value of all the six panels.

PV module type	K–S test statistical values			
	Minute Ramp up	Minute Ramp down	Hour Ramp up	Hour Ramp down
Single crystalline	0.12737	0.13863	0.12737	0.13863
Poly crystalline	0.1227	0.13408	0.1227	0.06655
Micro crystalline	0.12681	0.13329	0.05303	0.05696
Amorphous	0.11834	0.12585	0.05511	0.05639
CIS	0.11819	0.12814	0.05583	0.06585
HIT	0.13786	0.12498	0.05125	0.06044

The probability density function *f(x)* and Cumulative distribution function *F(x)* of Generalized Logistic Distribution are given by

7 $$f(x) = \left\{ {\begin{array}{*{20}l} {\frac{{(1 + kz)^{ - 1 - 1/k} }}{{\sigma \left( {1 + \left( {1 + kz} \right)^{ - 1/k} } \right)^{2} }}} \hfill & {k \ne 0} \hfill \\ {\frac{{\exp \left( { - z} \right)}}{{\sigma \left( {1 + \exp \left( { - z} \right)} \right)^{2} }}} \hfill & {k = 0} \hfill \\ \end{array} } \right.$$

and

8 $$F(x) = \left\{ {\begin{array}{*{20}l} {\frac{1}{{1 + \left( {1 + k\,z} \right)^{ - 1/k} }}} \hfill & {k \ne 0} \hfill \\ {\frac{1}{{1 + \exp \left( { - z} \right)}}} \hfill & {\,k = 0} \hfill \\ \end{array} } \right.$$

where $z = \,\,\,\frac{x - \mu }{\sigma }$, *k* is continuous shape parameter, $\sigma$ is a continuous scale parameter ($\sigma$ > 0) ,$\mu$ is a continuous location parameter, and *x* is the changes in power output.

**Fig. 7:**
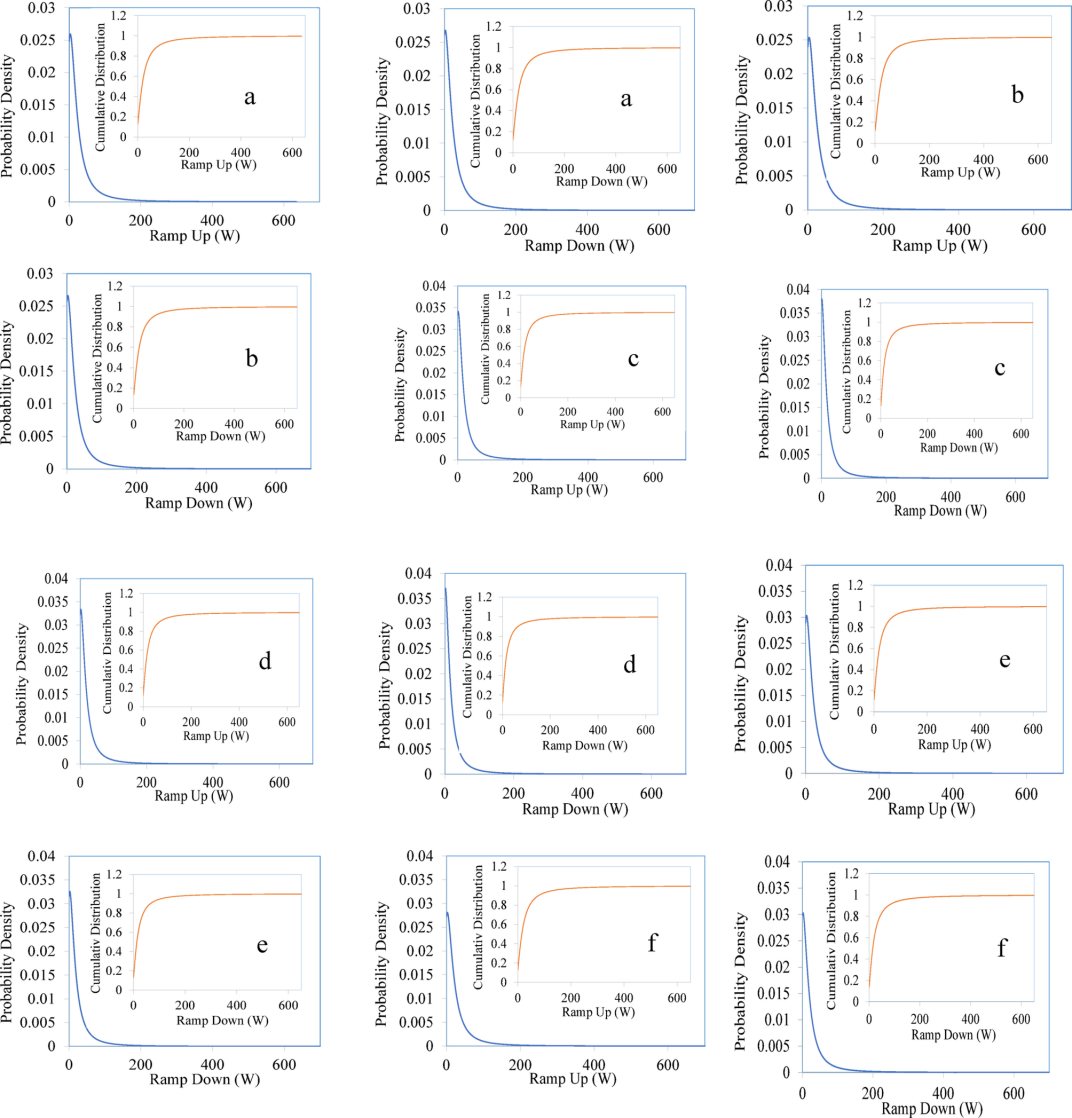
The probabilities for ramp up and ramp down of the panels (**a**) single crystalline (**b**) poly crystalline (**c**) micro crystalline (**d**) amorphous (**e**) CIS (**f**) HIT.

Table 3 presents the shape, scale, and location parameters for the continuous distributions of both minute and hourly power fluctuations. The continuous shape parameter k can give some indication on the ramp rate of the panels. Higher k values indicate sharper changes in power, indicating higher ramps. Substituting these values into Eqs. 7 and 8 yields the probability density and cumulative distribution of power ramps. Figure 7 shows these modeled probabilities for minute-wise ramp-up and ramp-down of the PV systems.

**Table 3:** Minute-wise and hourly generalized logistic distribution parameters.

PV module type	Fluctuations	Generalized logistic distribution parameters					
		Continuous shape parameter k		Continuous scale parameter $\sigma$		Continuous location parameter $\mu$	
		Ramp up	Ramp Down	Ramp up	Ramp Down	Ramp up	Ramp Down
Single crystalline	Minute-wise	0.59449	0.60501	14.06	13.801	16.117	15.257
	Hourly	0.15604	0.21691	61.107	57.34	146.96	115.25
Poly crystalline	Minute-wise	0.58644	0.59822	14.222	13.763	16.6	15.469
	Hourly	0.16042	0.21709	61.843	57.408	146.35	116.14
Micro crystalline	Minute-wise	0.63588	0.65516	11.338	10.488	12.603	11.313
	Hourly	0.13484	0.17492	58.56	53.271	145.09	118.15
Amorphous	Minute-wise	0.63388	0.65526	11.557	10.744	13.127	11.792
	Hourly	0.13789	0.18108	62.827	56.683	154.77	126.1
CIS	Minute-wise	0.60793	0.61729	12.233	11.557	14.192	12.99
	Hourly	0.13319	0.201171	60.27	56.658	149.54	117.79
HIT	Minute-wise	0.62253	0.63956	13.49	12.847	15.217	13.868
	Hourly	0.14408	0.1959	64.902	59.639	158.57	126.58

The 95% confidence interval for the ramps is presented in Table 4. The measured ramp ups and downs are compared with the corresponding modelled ramps in the table. Considering the shape parameter of the probability curve and looking at the measured and modelled ramps at 95% confidence interval, CIS based PV systems showed the lowest power ramps under the considered tropical environment. The highest power fluctuations are observed for the HIT based PV systems. The ramp-up and ramp-down characteristics are one of the important parameters for designing solar PV plants, as it indicates the capacity of the required energy storage solutions for the stable operation of the system^35^.

**Table 4:** 95% Confidence interval of ramp down fluctuation of six types solar PV module.

PV module type	95% Confidence interval of ramp up and down fluctuation(W)				
	Fluctuations	Actual Ramp up	Actual Ramp down	Modelled Ramp up	Modelled Ramp down
Single crystalline	Minute-Wise	182.1	185.55	128.63	127.9
	Hourly	372.36	351.18	375.35	351.57
Poly crystalline	Minute-Wise	182.1	182.9	128.7	126.38
	Hourly	380.96	353.55	379.1	352.81
Micro crystalline	Minute-Wise	175.7	174	110.7	105.49
	Hourly	355.39	322.43	356.77	323.23
Amorphous	Minute-Wise	178	177	112.76	108.29
	Hourly	383.23	346.13	382.96	346.58
CIS	Minute-Wise	163.6	158.75	114.59	109.54
	Hourly	367.57	347.08	366.83	345.61
HIT	Minute-Wise	202.48	204.5	129.04	125.84
	Hourly	395.89	362.56	396.6	364.15

### Predicting the ramping of the PV systems using machine learning

**Fig. 8:**
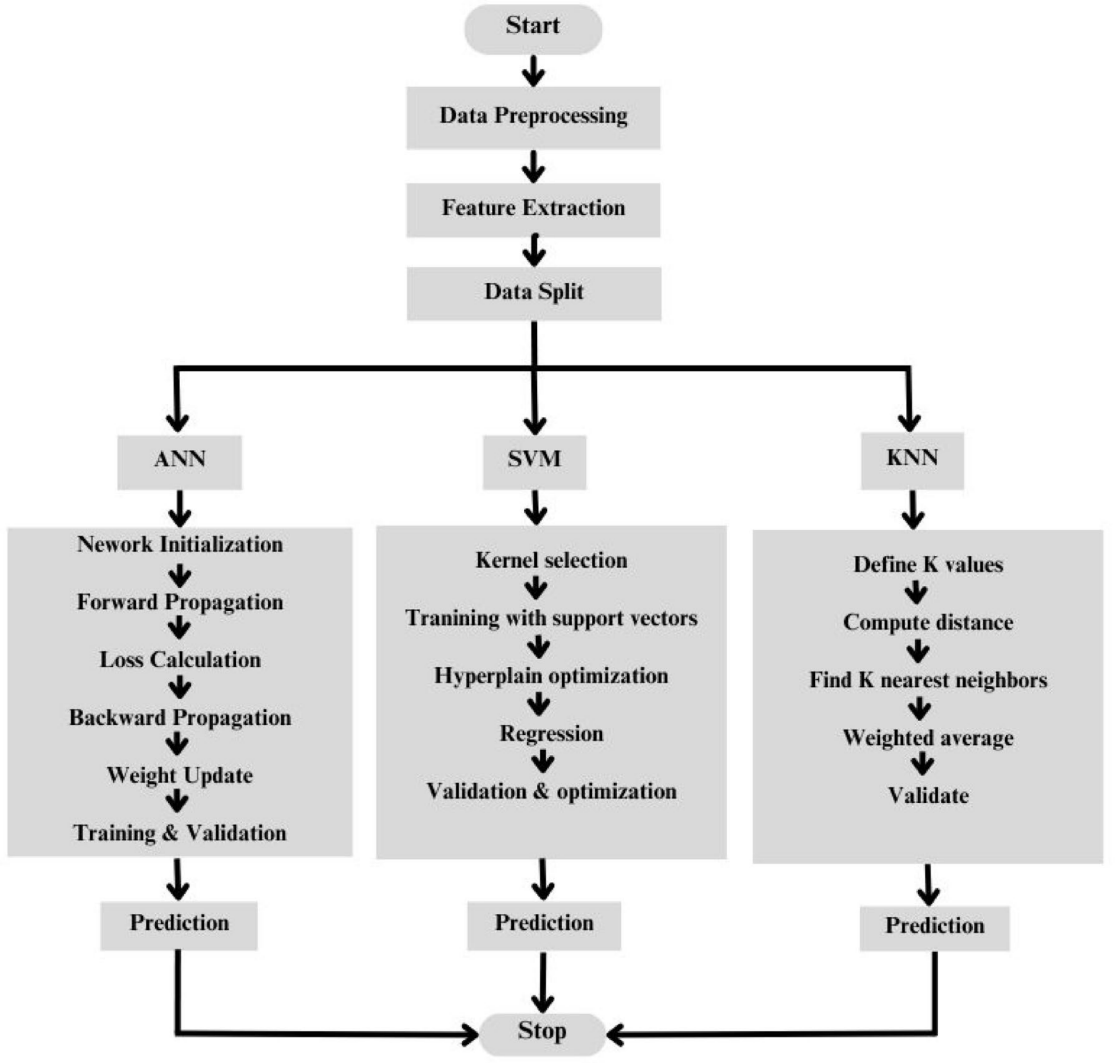
Various steps in developing the proposed machin learning models.

Models for predicting the possible power ramps from these PV systems, at prospective sites, are developed using machine learning methods like Artificial Neural Network (ANN), Support Vector Machine (SVM) and k-Nearest Neighbor (kNN) are shown as a flow chart in Fig. 8. General details of these models can be found in^36–40^. Three years of minute-wise and hourly data on the fluctuations in solar insolation and corresponding PV power fluctuations (Eq. 6) were used for developing these models. These are briefly explained in the following sections.

#### Artificial neural network (ANN)

Artificial Neural Network (ANN) is being widely used for modeling the performance of solar PV systems^19,36,37,41,42^. Among the various available network algorithms, Resilient Back Propagation with weight backtracking (RPROP +)^31^ is utilized for the current analysis. In RPROP + , a separate learning rate is used for each weight that can be changed during the training process in contrast with the traditional Back Propagation Network, where one learning rate is used for the entire training process. The weights are adjusted by the following rule

9 $$w_{j}^{(l + 1)} = w_{j}^{(l)} - \eta_{j}^{(l)} \cdot sign\left( {\frac{{\partial E^{(l)} }}{{\partial w_{j}^{(l)} }}} \right)$$

where $w_{j}^{(l)}$ *j*^*th*^ weight at *l*^*th*^ iteration step, $\upeta _{j}^{(l)}$ is *j*^*th*^ learning rate at* l*^*th*^ iteration step, $\frac{{\partial E^{(l)} }}{{\partial w_{j}^{(l)} }}$ is the partial derivative of error function to weight at *l*^*th*^ iteration step. Error function applied in this model is the Sum of Squared Error (SSE) that measures the deviation from the actual output during the training time.

To predict the model an output layer with single node and two hidden layers with five and four nodes respectively are used. The number of layers and node has been chosen based on the root mean square errors. These layers are activated by tangent hyperbolic transfer function. The threshold value for the partial derivative of the error function is set as 0.01 which is considered as a stopping criterion of the entire process. This Neural network model learns an approximation function between irradiance and power fluctuation that can be used to predict power fluctuation with respect to new irradiance data set.

#### Support vector machine (SVM)

Support Vector Machine (SVM) is another powerful machine learning method that performs well with both linear and nonlinear classification and regression problems. If the inputs are nonlinearly separable, it is first mapped into higher dimensional space, using kernel function and then a linear model on that feature space is constructed.

By estimating the accuracy of minute- wise and hourly solar ramp predictions, SVM with radial kernels was chosen for the analysis. The model features, optimized by tuning the errors, include Cost *C* = (0.1, 1, 100), gamma $\gamma$ = (0.01, 1).

#### k-nearest neighbor (kNN)

The k-Nearest Neighbor is the simplest machine-learning method that can be used for both classification and regression technique. The method for developing the algorithms for kernel-based K-NN is described in the steps below [22–24].

1. Consider $G = \left\{ {z,y} \right\}_{i = 1}^{N}$ be training dataset where *z* is the variation in irradiance, *y* is the power fluctuation, let* z* be a new data whose power fluctuation must be predicted.

2. Find the *k* + *1* nearest neighbors for new data set *z* according to a Minkowski distance function

10 $$d(z_{i} ,z_{j} ) = \left( {\sum\limits_{s = 1}^{p} {\left| {z_{is} - z_{js} } \right|}^{q} } \right)^{\frac{1}{q}}$$

where *q* is the order between two data points *z*_*i*_ and *z*_*j*_ , here it is set it as two; *p* is the total number of training data points.

3. Transform the distance with optimal kernel function into weights *w*_*i*_ such that

11 $$w_{i} = \left[ {\left( {\frac{2(d + 4)}{{d + 2}}} \right)^{{\left( {\frac{d}{d + 4}} \right)}} k} \right]$$

where *d* is the Minkowski distance, *k* is set it as seven which selected iteratively.

Compute the weighted averaged* k* nearest neighbor for predicting the output of new data set *z.* For the new dataset, the number of nearest neighbors *(k)* used to compute the distance function is set to 7*.*

The performance of the models developed using the above machine learning methods is assessed by comparing the modeled ramps with the ramps calculated from the actual power measurements. The prediction errors are quantified using matrices like Mean Absolute Error (MAE), Root Mean Square Error (RMSE), and Normalized Root Mean Square Error (NRMSE). These errors for the minute-wise and hourly ramp predictions are presented in Table 5 and Table 6 respectively. The errors of the models vary with the machine learning methods and the type of panels. For example, for the minute-wise predictions, SVM performs well for single crystalline, whereas ANN-based models were found better for polycrystalline, HIT, CIS, amorphous and microcrystalline panels. Similarly, when comparing the hourly prediction results, models with ANN show the best results for Single Crystalline silicon, Poly Crystalline silicon, Amorphous PV module and CIS, whereas SVM is found better for Micro Crystalline silicon and HIT PV modules. Nevertheless, in spite of these slight variations in the accuracies, in general, the solar ramp prediction models with all the machine learning techniques could achieve impressive accuracies.

**Table 5:** Error analysis of the minute-wise models based on machine learning.

Method	Error	PV module type					
		Single crystalline	Poly crystalline	Micro crystalline	Amorphous crystalline	CIS	HIT
ANN	RMSE	30.699	37.326	60.955	53.590	53.321	69.797
	NRMSE	0.023	0.028	0.046	0.039	0.039	0.049
	MAE	14.599	16.879	24.566	21.016	22.381	28.982
SVM (Radial)	RMSE	30.682	37.628	61.317	53.973	53.595	70.666
	NRMSE	0.023	0.028	0.047	0.040	0.039	0.050
	MAE	14.420	16.811	24.557	21.056	22.388	29.003
k-NN	RMSE	33.467	41.837	67.219	59.322	60.101	77.133
	NRMSE	0.025	0.027	0.043	0.036	0.037	0.044
	MAE	17.50228	21.87407	32.34022	27.29829	31.07534	36.8651

**Table 6:** Error analysis of the hourly models based on machine learning.

Method	Error	PV module type					
		Single crystalline	Poly crystalline	Micro crystalline	Amorphous crystalline	CIS	HIT
ANN	RMSE	30.980	32.838	29.608	27.377	31.506	38.103
	NRMSE	0.025	0.023	0.023	0.019	0.023	0.027
	MAE	24.304	25.835	21.953	20.822	23.591	29.093
SVM (Radial)	RMSE	31.692	33.014	29.212	28.343	32.257	38.039
	NRMSE	0.025	0.024	0.023	0.020	0.023	0.027
	MAE	23.784	25.677	21.366	20.134	24.184	28.509
k-NN	RMSE	38.182	38.107	35.244	33.903	36.904	43.665
	NRMSE	0.030	0.027	0.027	0.024	0.027	0.031
	MAE	28.644	29.426	25.676	24.636	28.060	33.078

**Fig. 9:**
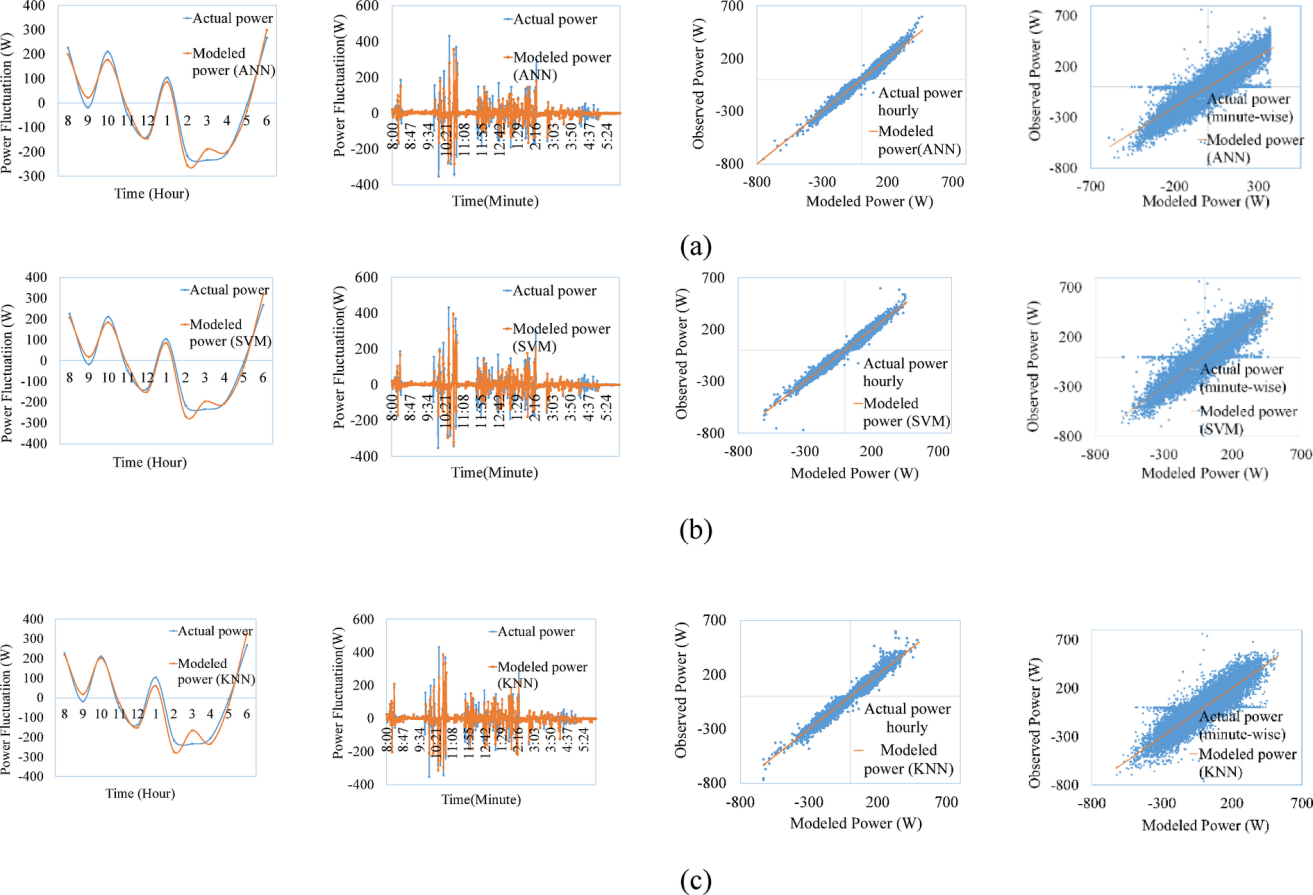
Comparison between ramps predicted using the machine learning method and the actual power ramp measurements of single crystalline in a day and all year (**a**) ANN, (**b**) SVM (**c**) KNN.

**Fig. 10:**
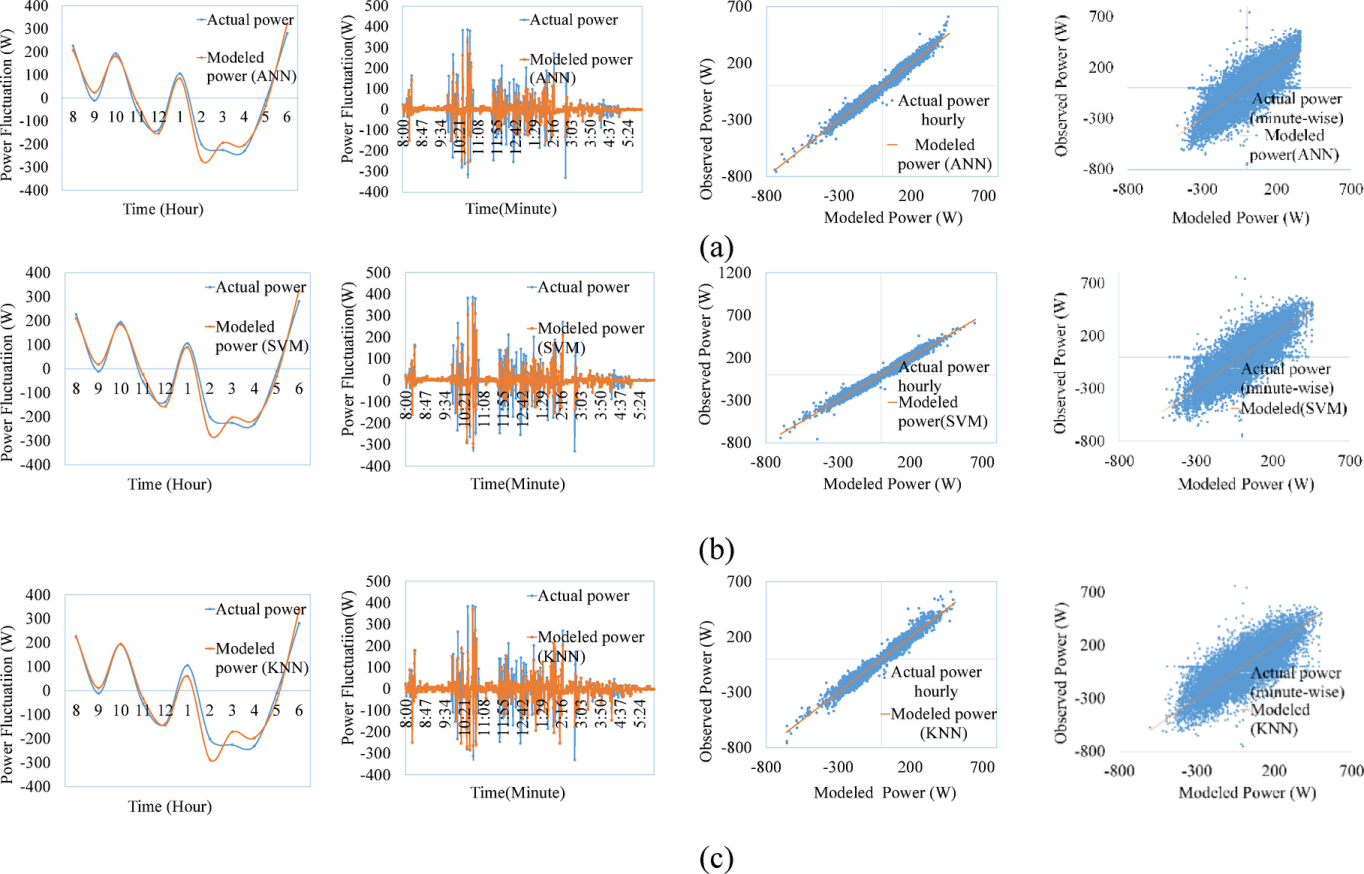
Comparison between ramps predicted using the machine learning method and the actual power ramp measurements of poly crystalline in a day and all year (**a**) ANN, (**b**) SVM, (**c**) KNN.

**Fig. 11:**
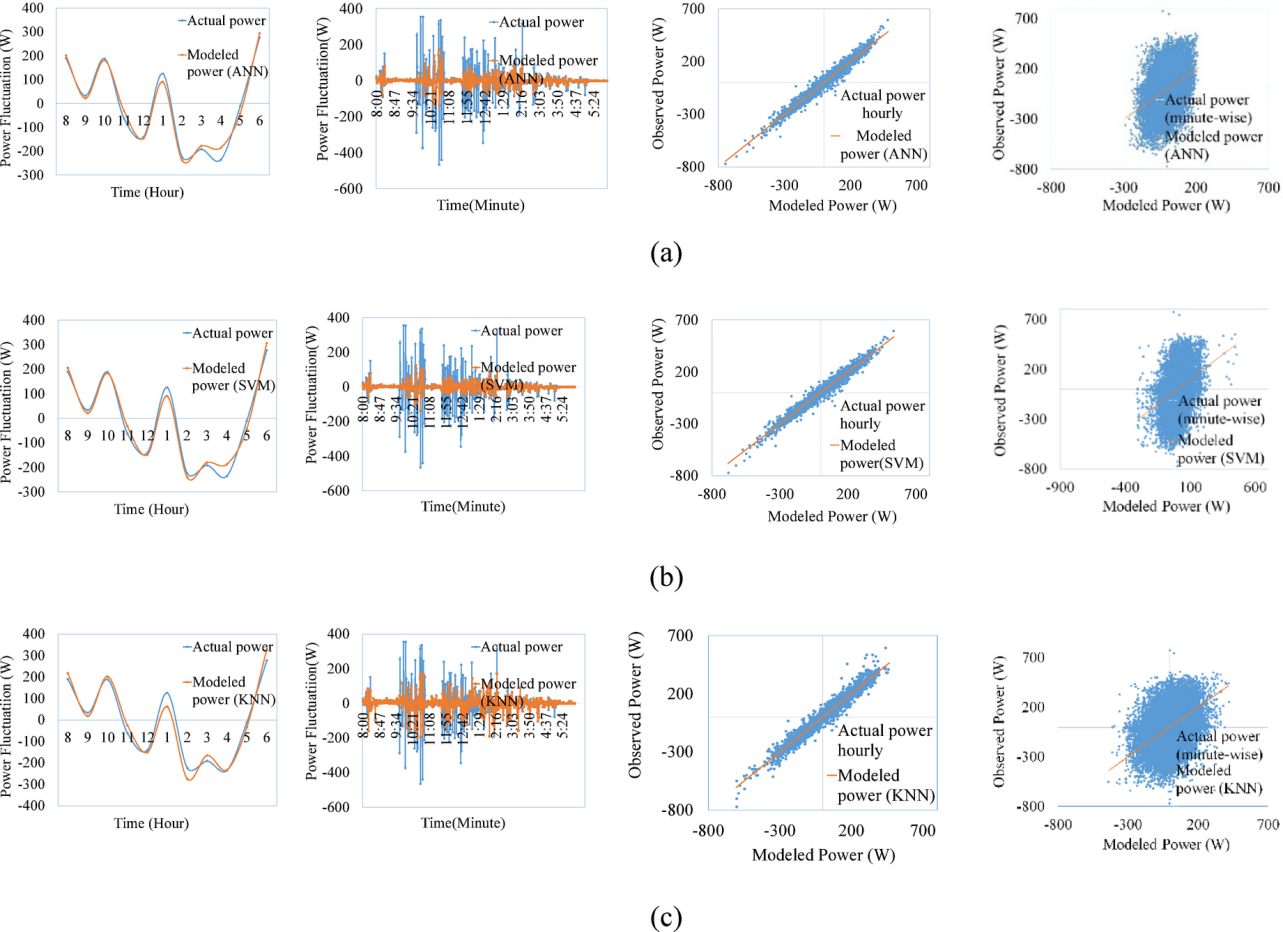
Comparison between ramps predicted using the machine learning method and the actual power ramp measurements of micro crystalline in a day and all year (**a**) ANN, (**b**) SVM, (**c**) KNN.

**Fig.12:**
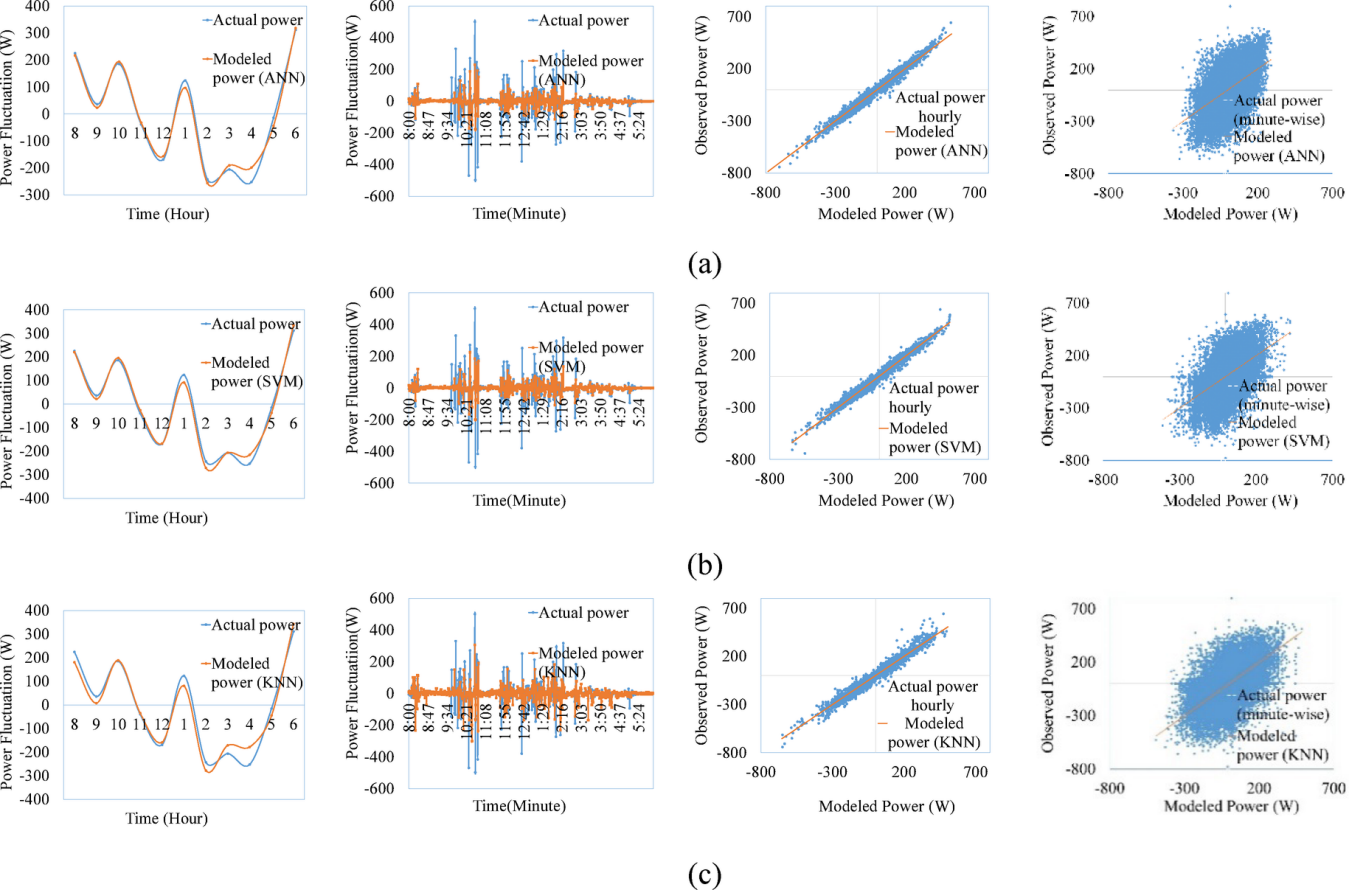
Comparison between ramps predicted using the machine learning method and the actual power ramp measurements of amorphous silicon in a day and all year (**a**) ANN, (**b**) SVM (**c**) KNN.

**Fig.13:**
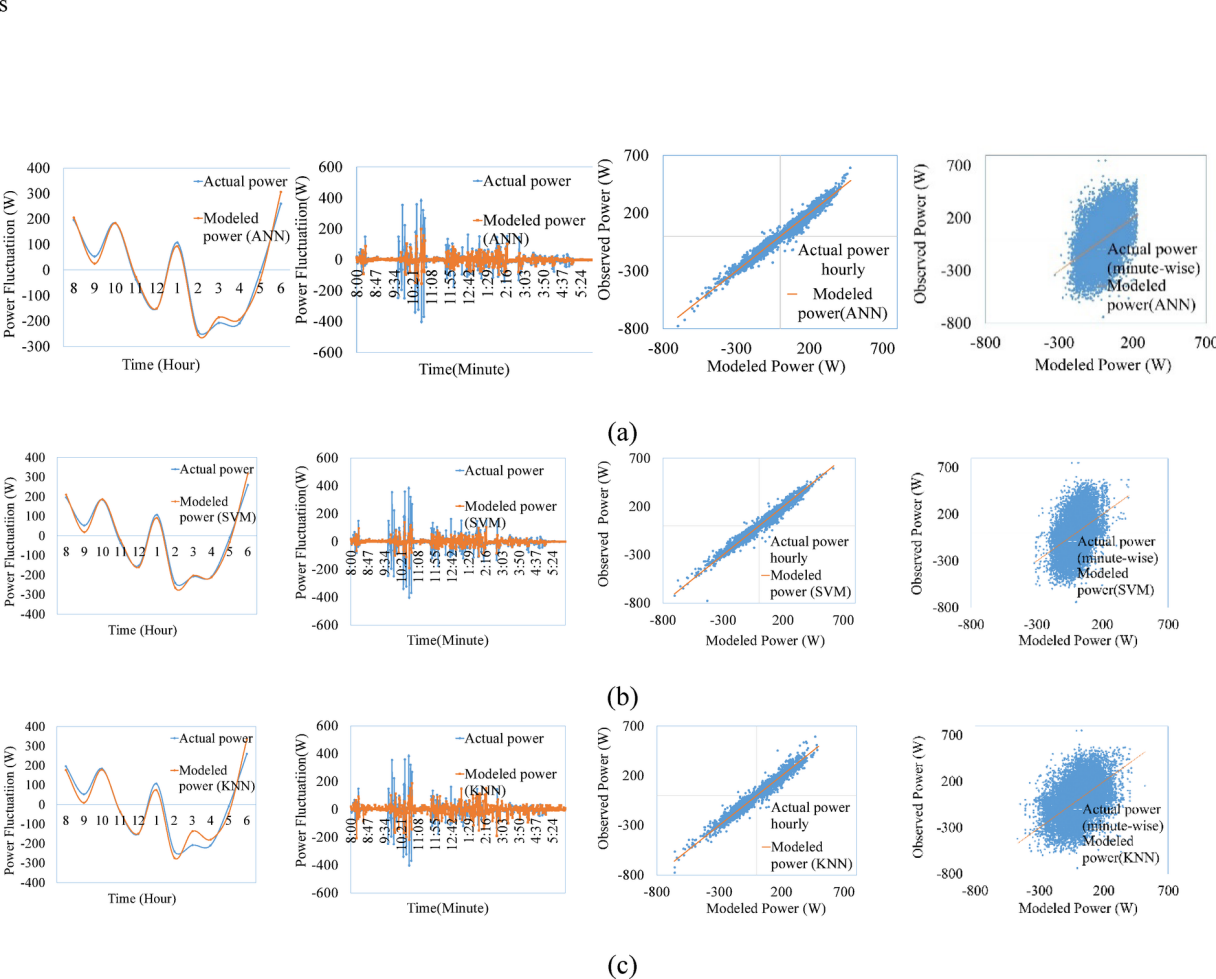
Comparison between ramps predicted using the machine learning method and the actual power ramp measurements of CIS in a day and all year (**a**) ANN, (**b**) SVM, (**c**) KNN.

**Fig. 14:**
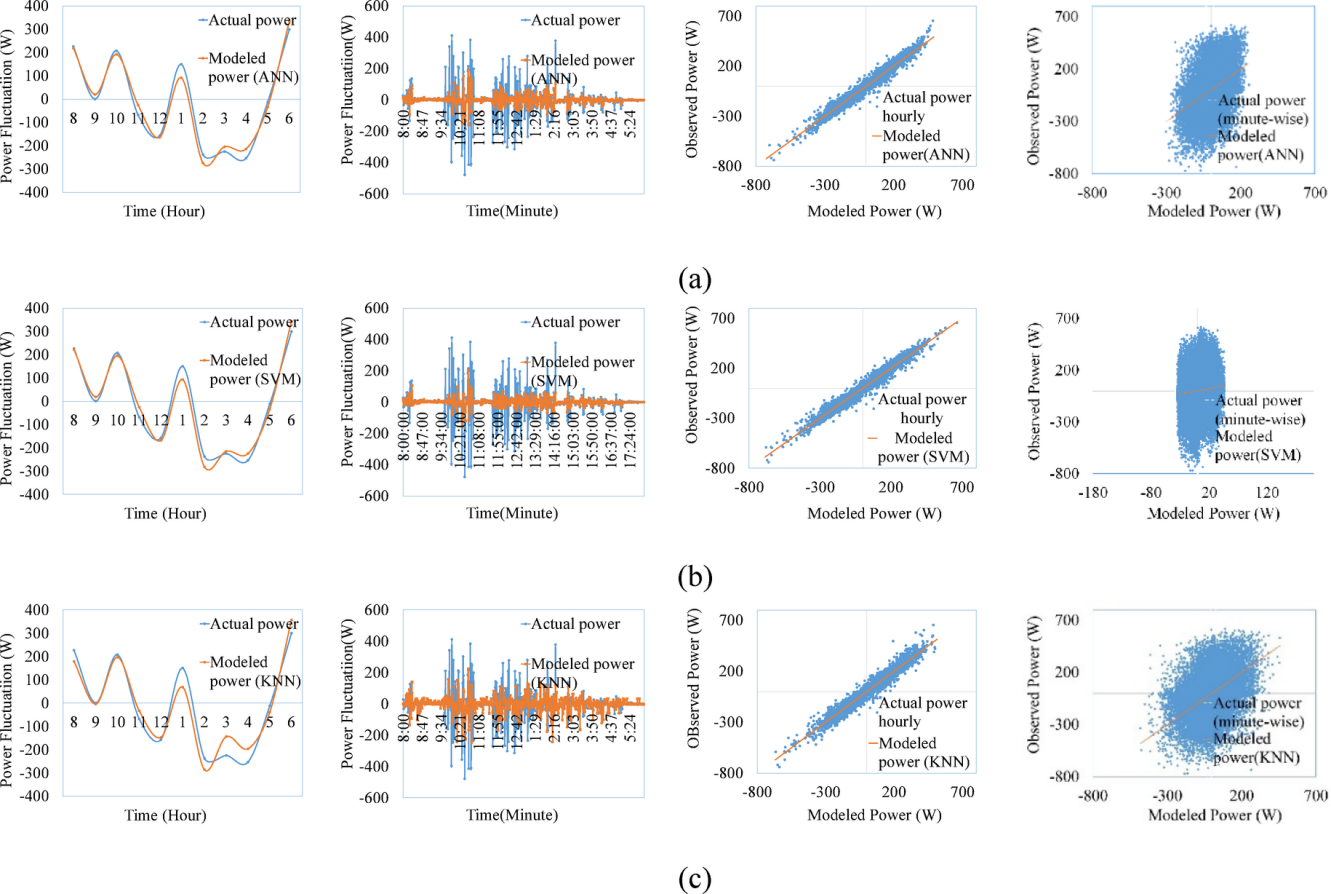
Comparison between ramps predicted using the machine learning method and the actual power ramp measurements of HIT in a day and all year (**a**) ANN, (**b**) SVM, (**c**) KNN.

Figures 9, 10, 11, 12, 13, 14 compare the power ramps of the systems estimated using different machine learning models with actual measurements. The accuracy of these estimations varies depending on the modeling approach. ANN-based predictions are more accurate due to their ability to learn complex nonlinear patterns in solar power variations. This is achieved through the multi-layer perceptron architecture and the RPROP + algorithm, which efficiently updates and optimizes weights and biases. Support Vector Machines (SVM) also perform well, due to it’s kernel trick, by which the nonlinear relationships are efficiently map the varying irradiance conditions with the PV ramp outputs. In contrast, k-Nearest Neighbors (kNN) exhibits lower accuracy due to its simplistic modeling approach, which struggles to capture the power dynamics of PV systems. Additionally, kNN is sensitive to noisy data and cannot effectively model high-dimensional relationships. ANN and SVM-based models can also generalize from historical data and mitigate the adverse effects of outliers using soft margin techniques (SVM) or resilient backpropagation (ANN).

The ramp models developed in this study have substantial practical value for the design and management of prospective solar PV plants. By integrating these models with time series data on solar insolation variations at the site, designers gain insights into expected fluctuations in power output from the PV systems. Understanding these ramp patterns prior to the construction and integration of solar farms enables developers to optimally size the system and arrange alternative generation options to effectively manage load during ramp-down periods. Additionally, these insights assist design engineers in determining the necessary capacity of storage solutions for the plant. The hourly predictions from the models can serve as valuable tools for developing optimal dispatch strategies for power systems integrated with PVs. Furthermore, when paired with solar insolation forecasts for the plant location, these models enable system owners to accurately commit their production across various time scales, thereby facilitating efficient participation in energy markets.

## Conclusions

This paper presents a performance analysis of six different solar PV systems in a tropical environment, based on data from a 1.2 MW experimental solar farm. The PV technologies analysed include single crystalline silicon (sc-Si), polycrystalline silicon (pc-Si), microcrystalline silicon (mc-Si), amorphous silicon (a-Si), copper indium selenium (CIS), and heterojunction with an intrinsic thin layer (HIT). Performance evaluation was conducted using key metrics such as Array Yield, Reference Yield, Capture Loss, Performance Ratio, and Efficiency Ratio.

Among these technologies, amorphous silicon-based systems exhibited the highest performance, achieving an array yield of 4.81 h/day, whereas CIS-based modules recorded the lowest array yield at 3.12 h/day. Capture losses were minimal for HIT (18.06%) and a-Si (18.22%), while sc-Si experienced the highest loss (27.15%). Similarly, HIT and a-Si achieved the highest Performance Ratios (0.80 and 0.81, respectively), whereas sc-Si had the lowest (0.69).

The ramp-up and ramp-down behaviours of these systems were analysed using probabilistic modeling, revealing that all six PV technologies followed the Generalized Logistic Distribution. HIT-based PV systems exhibited the highest power fluctuations, with a 95th percentile hourly ramp-up of 396.6 W and a ramp-down of 364.15 W. In contrast, CIS-based systems had the lowest ramping rates (95th percentile hourly ramp-up: 356.77 W, ramp-down: 323.23 W). These findings provide critical insights for the the appropriate sizing of energy storage solutions for the PV systems.

To predict the ramping behaviour of these PV systems, machine learning models based on Artificial Neural Networks (ANNs), Support Vector Machines (SVMs), and k-Nearest Neighbors (k-NN) were developed. All models achieved high accuracy, exceeding 96%. These predictive models, when integrated with real-time solar insolation forecasts, can help to forecast power ramps, thereby facilitating the efficient grid and market integration of large-scale solar PV power plants under similar operating environments.

## Data Availability

All data generated or analyzed during this study are included in this published article.
